# Preclinical Characterization of Efficacy and Pharmacodynamic Properties of Finotonlimab, a Humanized Anti-PD-1 Monoclonal Antibody

**DOI:** 10.3390/ph18030395

**Published:** 2025-03-12

**Authors:** Yunqi Yao, Xiaoning Yang, Jing Li, Erhong Guo, Huiyu Wang, Chunyun Sun, Zhangyong Hong, Xiao Zhang, Jilei Jia, Rui Wang, Juan Ma, Yaqi Dai, Mingjing Deng, Chulin Yu, Lingling Sun, Liangzhi Xie

**Affiliations:** 1 Beijing Engineering Research Center of Protein and Antibody, Sinocelltech Ltd., Beijing 100176, China; yunqi_yao@sinocelltech.com (Y.Y.); xiaoning_yang@sinocelltech.com (X.Y.); jing_li3@sinocelltech.com (J.L.); erhong_guo@sinocelltech.com (E.G.); huiyu_wang@sinocelltech.com (H.W.); chunyun_sun@sinocelltech.com (C.S.); xiao_zhang@sinocelltech.com (X.Z.); jilei_jia@sinocelltech.com (J.J.); rui_wang@sinocelltech.com (R.W.); juan_ma@sinocelltech.com (J.M.); yaqi_dai@sinocelltech.com (Y.D.); mingjing_deng@sinocelltech.com (M.D.); chulin_yu@sinocelltech.com (C.Y.); lingling_sun@sinocelltech.com (L.S.); 2 State Key Laboratory of Medicinal Chemical Biology, Tianjin Key Laboratory of Protein Sciences, Cancer Biology Center, Frontiers Science Center for New Organic Matter, College of Life Sciences, Nankai University, Tianjin 300071, China; hongzy@nankai.edu.cn; 3 Beijing Key Laboratory of Monoclonal Antibody Research and Development, Sino Biological Inc., Beijing 100176, China; 4 Cell Culture Engineering Center, Chinese Academy of Medical Sciences & Peking Union Medical College, Beijing 100005, China

**Keywords:** finotonlimab, anti-PD-1 antibody, preclinical, characterization, pharmacodynamics

## Abstract

**Background/Objectives:** Finotonlimab (SCTI10A) is a humanized anti-PD-1 antibody tested in Phase III trials for several solid tumor types. **Methods**: This study characterized the in vitro and in vivo efficacy, Fc-mediated effector function, and non-clinical PK/PD properties of finotonlimab. **Results**: The results demonstrated that finotonlimab is effective in stimulating human T cell function in vitro and exhibits marked antitumor efficacy in vivo using both PD-1-humanized and PBMC-reconstructed mouse models. Additionally, finotonlimab exhibited minimal impact on the activation of effector cells via Fc receptor-dependent pathways, potentially facilitating PD-1^+^ T cell killing. In cynomolgus monkeys, finotonlimab exhibited a nonlinear pharmacokinetic (PK) profile in a dose-dependent manner, and a receptor occupancy rate of approximately 90% was observed at 168 h following a single administration of 1 mg/kg. Finotonlimab’s PK profile (especially C_max_) was better than that of marketed antibodies. Following a 13-week successive administration of finotonlimab, a pharmacodynamic analysis revealed that a sustained mean receptor occupancy of PD-1 molecules on circulating T cells remained at or above 93% for up to 8 weeks, even at a dose of 3 mg/kg, and that there were higher antibody accumulations in different dose groups. **Conclusions**: Taken together, the preclinical findings are promising and provide the groundwork for evaluating the efficacy and pharmacodynamic characteristics of finotonlimab in clinical trials.

## 1. Introduction

Targeting programmed cell death protein-1 (PD-1) and its ligand (PD-L1) has emerged as a pivotal immunotherapy strategy, profoundly transforming the therapeutic landscape for patients with cancer. In clinical applications, anti-PD-1 antibodies have demonstrated significant benefits, including increased median overall survival and progress-free survival across multiple cancer types [1,2,3].

PD-1 is an inhibitory immune checkpoint receptor [4,5,6], and its expression is observed on activated T cells, natural killer cells (NKs), B lymphocytes [7], macrophages, dendritic cells (DCs) [8], and monocytes [9] as an immune suppressor for both adaptive and innate immune responses. Engagement of PD-1 by its ligands PD-L1 [10] or PD-L2 [11,12] leads to the exhaustion of T cell function and immune tolerance in the tumor microenvironment. Therefore, blockade of the PD-1 pathway was considered a breakthrough to inhibit tumor immune escape and enhance T cell function to destroy the cancer cells, thereby achieving substantial antitumor effects [13,14].

Both preclinical and clinical investigations have shown that antibodies targeting PD-L1 and blocking receptor–ligand interactions could be effective in activating T cell function and immune response [2]. However, variations in the binding footprints between PD-1 antibodies and PD-1 have been observed, resulting in different binding affinities and biological activities [15,16]. Additionally, the constant region of an antibody exerts secondary pharmacodynamic effects through interactions with FcγRs or activation of a complement cascade, which also plays a significant role [17]. Consequently, anti-PD-1 antibodies with diverse functional and pharmacokinetic characteristics offer potential for different dosing requirements, safety considerations, and personalized treatment approaches for specific individuals and cancer types.

Finotonlimab (SCTI10A) is a high-affinity humanized antibody screened from an antibody library by phage displaying. Finotonlimab is currently under Phase III investigations for multiple solid tumor types as a monotherapy and in combination with other drugs, including squamous-cell NSCLC (NCT04171284), HCC (NCT04560894), and HNSCC (NCT04146181). A phase I trial (NCT03821363), which enrolled 274 patients, demonstrated the safety of different doses of SCT-I10A and its long PK profile and efficacy in different tumor types. In a phase III trial (NCT04146402) enrolling 370 patients, SCT-I10A combined with chemotherapy prolonged the median overall survival (OS) of patients with HNSCC to 14.1 months and reduced the risk of death by 27% [18]. In particular, the SCT-I10A regimen reduced the risk of death by 50% in patients with a combined positive score (CPS) ≥ 20. In a phase III trial (NCT04560894) involving 346 patients with HCC, SCT-I10A combined with SCT510 (bevacizumab biosimilar) prolonged median OS to 22.1 months and reduced the risk of death by 40% compared to sorafenib. Overall, SCT-I10A shows encouraging therapeutic efficacy in recurrent or metastatic head and neck squamous cell carcinoma (HNSCC) and advanced hepatocellular carcinoma (HCC).

Herein, the in vitro and in vivo pharmacology, Fc-mediated effector functions, and pharmacokinetic/pharmacodynamic (PK/PD) profile in cynomolgus monkeys of finotonlimab were comprehensively characterized. All the promising results have served as a crucial foundation for ongoing clinical assessments.

## 2. Results

### 2.1. High Affinity and Binding Specificity of Finotonlimab to hPD-1

**Figure 1:**
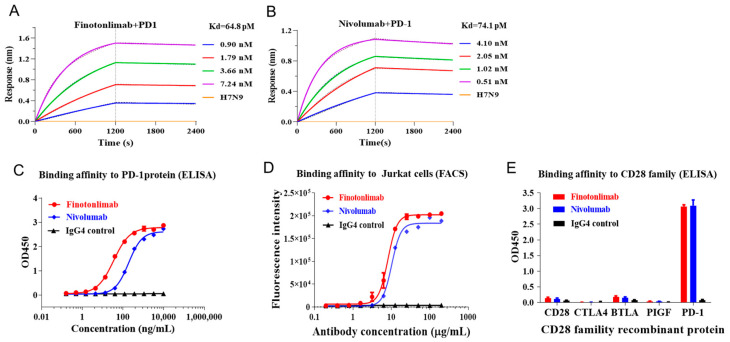
The affinity and binding specificity of finotonlimab to hPD-1 were evaluated using Octet and cell-based assays. (**A**) Bio-layer interferometry was employed to determine the binding avidity of finotonlimab (**A**) and nivolumab (**B**) to hPD-1. (**C**) ELISA was performed to assess the binding affinity of finotonlimab and nivolumab to hPD-1 protein (n = 3). (**D**) The binding affinity of finotonlimab and nivolumab to PD-1 -overexpressed Jurkat cells (n = 3). (**E**) The binding affinity of finotonlimab and nivolumab to homologous proteins, such as CD28 and CTLA-4 (n = 3).

The binding kinetics (association and dissociation patterns) of finotonlimab with human PD-1 protein were assessed using bio-layer interferometry. The results indicated that finotonlimab demonstrated high avidity to hPD-1, with a K_D_ value of 6.48 × 10^−11^ M and a lower dissociation rate (1.95 × 10^−5^ s^−1^) compared to nivolumab (5.12 × 10^−5^ s^−1^) (Figure 1A,B, Table 1). The binding ability of finotonlimab to both hPD-1 protein and hPD-1-engineered Jurkat cells exhibited a concentration-dependent pattern, which was comparable to nivolumab (Figure 1C,D). The EC_50_ values of hPD-1 protein for finotonlimab and nivolumab were 34.5 ng/mL and 190 ng/mL, respectively, with a 5.51-fold reduction in EC_50_ observed for finotonlimab. Additionally, finotonlimab exhibited selective binding to PD-1, with no detectable interaction with other CD28-homologous proteins, such as CD28, CTLA-4, BTLA, and PIGF (Figure 1E).

### 2.2. Epitope of Finotonlimab Overlaps with PD-L1/PD-L2 Binding Sites

**Figure 2:**
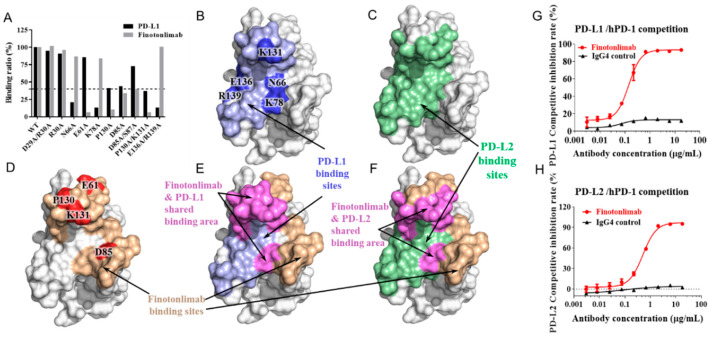
Finotonlimab blocks the binding of PD-1 ligands to PD-1 and its epitope mapping. (**A**) Binding ratio of PD-L1/finotonlimab with various PD-1 mutants. (**B**) PD-L1 binding sites on PD-1 are depicted in light blue, while binding sites identified through mutation experiment are highlighted in blue. (**C**) PD-L2 binding sites on PD-1 are shown in green. (**D**) Epitopes of finotonlimab, identified by docking simulation and mutation experiment, are displayed in light red and red, respectively. (**E**) Binding sites of both PD-L1 and finotonlimab on PD-1 are illustrated, with overlapping regions highlighted in magenta. (**F**) Binding sites of both PD-L2 and finotonlimab i on PD-1 are shown, with overlapping regions in magenta. Competitive inhibition rates of finotonlimab against the PD-L1/hPD-1 and PD-L2/hPD-1 are presented in (**G**,**H**), respectively (n = 3).

A mutation study was conducted to elucidate the epitope of finotonlimab and the binding sites of PD-L1 (Figure 2A). The results showed that specific PD-1 mutants (N66, K78, K131, and E136/R139) had markedly diminished binding abilities (<40%) to PD-L1. These findings are consistent with the analysis of the crystal structure of PD-1 and PD-L1 complex (PDB ID 4ZQK), demonstrating the reliability of our mutation-based method (Figure 2B). Similarly, the structural analysis of the PD-1 and PD-L2 complex (PDB ID 6UMT) demonstrated a comparable pattern in the binding sites of PD-L2 on PD-1 as observed for PD-L1 (Figure 2C).

By utilizing these specific mutations in PD-1, we identified E61, D85, and P130/K131 as crucial binding sites for finotonlimab (Figure 2D). The PD-1 and finotonlimab complex structure of PD-1 and finotonlimab was constructed through docking the homology model of finotonlimab with the PD-1 structure utilizing Discovery Studio, while considering the identified binding sites. In its complex structure with PD-1, the epitope of finotonlimab extensively overlaps with the PD-L1 binding sites on PD-1, covering approximately 44% of the PD-L1 binding area (Figure 2E). This indicates that finotonlimab has the capacity to effectively outcompete PD-L1 for binding to PD-1. Similarly, finotonlimab also competed effectively with PD-L2 for PD-1 binding, occupying around 32% of the PD-L2 binding area (Figure 2F).

Compared to an IgG4 isotype control, finotonlimab efficiently blocked both PD-1/PD-L1 and PD-1/PD-L2 binding, exhibiting half-maximal inhibitory concentration (IC_50_) values of 0.142 and 0.505 μg/mL, respectively (Figure 2G,H). In a cell-based functional competition assay on Jurkat cells, finotonlimab demonstrated potent inhibition of the interaction between PD-L1 and PD-1 with an IC_50_ value of approximately 1.8 μg/mL, while nivolumab had an IC_50_ value of 2.5 μg/mL (Supplementary Figure S1).

### 2.3. In Vitro Effects on the Function of T Cell Activation by Finotonlimab

**Figure 3:**
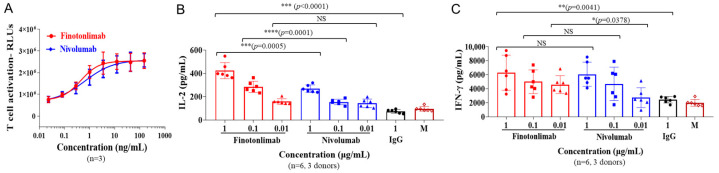
The functional activity of finotonlimab in T cell-based assays. (**A**) T cell activation was evaluated by measuring the luciferase signal level in Jurkat-NFAT-Luc2p-PD-1 reporter cells (n = 3). The assay was based on the interaction between effector cells (Jurkat-NFAT-Luc2p-PD-1) and target cells (CHO-K1-PD-L1-CD3E). In the MLR assay, levels of IL-2 (**B**) and IFN-γ (**C**) were quantified using ELISA (CD4^+^ T cells and DC were isolated from the PBMCs of different donors and co-cultured in the presence of anti-PD-1 antibodies or isotype controls at various concentrations for 5 days (the PBMCs were isolated from 3 donors). * *p* < 0.05, ** *p* < 0.01, *** *p* < 0.001, **** *p* <0.0001 and NS (no statistical significance).

The influence of finotonlimab on T cell function was evaluated using a T cell reporter assay and mixed lymphocyte reaction (MLR). To demonstrate the effect of T cell activation, we employed a PD-1/PD-L1 blocking bioassay system comprising Jurkat-PD-1-NFAT-Luc cells (Jurkat cell stably expressing human PD-1 and an NFAT (nuclear factor of activated T cell)-driven luciferase reporter), and CHOK1-PD-L1-TCRa cells (CHO-K1 line expressing human PD-L1 and an engineered TCR activator). As shown in Figure 3A, the functional activation of finotonlimab was assessed by quantifying the luciferase signal intensity in the reporter cells. The results indicated that finotonlimab exhibited potent concentration-dependent activity in promoting T cell response comparable to nivolumab.

We subsequently assessed the functional antagonist activity of finotonlimab in enhancing the response of primary human CD4^+^ T cell in an in vitro T cell MLR assay. Finotonlimab significantly potentiated T cell activation, as evidenced by elevated levels of interleukin IL-2 and IFN-γ production. The augmented activation of CD4^+^ T cells mediated by anti-PD-1 antibodies was observed to be concentration dependent (Figure 3B,C). Notably, at higher dose levels, finotonlimab stimulated significantly higher levels of IL-2 compared to nivolumab, while exhibiting similar effects at lower dose levels. Specifically, the concentrations of IL-2 induced by finotonlimab and nivolumab at 0.01, 0.1, and 1 μg/mL were measured as follows: 159 ± 24 vs. 143 ± 37 pg/mL; 286 ± 48 vs. 155 ± 24 pg/mL; 424 ± 69 vs. 269 ± 32 pg/mL, respectively (Figure 3B). The fold differences between the IL-2 levels induced by finotonlimab and nivolumab at these three concentrations were calculated as follows: 1.11 (0.01 μg/mL), 1.84 (0.1 μg/mL), and 1.57 (1 μg/mL) (Figure 3B). Regarding IFN-γ stimulation, finotonlimab showed comparable effects to nivolumab in terms of high-dose-level induction, yet it significantly outperformed nivolumab in terms of low-dose-level induction. The concentrations of IFN-γ triggered by finotonlimab and nivolumab at 0.01, 0.1, and 1 μg/mL were as follows: 4592 ± 1272, 5011 ± 1694, 6290 ± 2505 pg/mL for finotonlimab and 2742 ± 1402, 4707 ± 2375, 6070 ± 1728 pg/mL for nivolumab (Figure 3C).

### 2.4. Finotonlimab Exhibited Antitumor Activity in Mouse Xenograft Models

**Figure 4:**
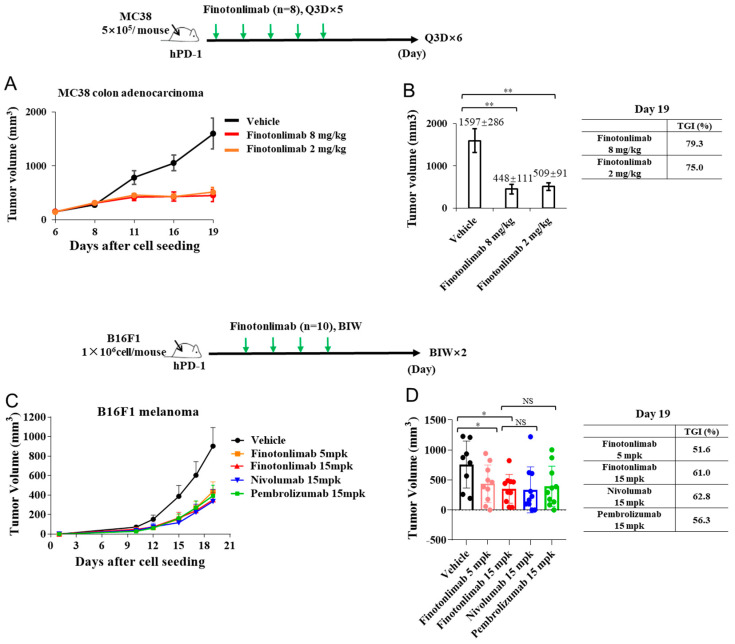
The antitumor efficacy of finotonlimab monotherapy was assessed in hPD1 humanized mouse models. Tumor volume (**A**) and percentage of tumor growth inhibition (TGI %) (**B**) in humanized mice with subcutaneous MC38 colon adenocarcinoma treated with finotonlimab. The dosing regimen was every three days (Q3D) for five doses (n = 8/group). Tumor volume (**C**) and TGI % (**D**) in the humanized mouse model of B16F1 melanoma treated with finotonlimab. The dosing schedule was twice a week (BIW) or four doses (n = 10/group). * *p* < 0.05, ** *p* < 0.01 and NS (no statistical significance).

The antitumor efficacy of finotonlimab was evaluated in a PD-1 humanized mouse model harboring MC38 tumors (Figure 4A,B). Mice bearing tumors were administered either vehicle control or finotonlimab at doses of 2 and 8 mg/kg every 3 days for a total of 5 treatments. Tumor sizes were assessed biweekly until 19 days after the initial treatment. Comparison of the relative tumor volume between the finotonlimab-treated group and the vehicle group indicated its ability to inhibit tumor growth. On the 19th day, the mean tumor volume in the vehicle-treated group reached approximately 1597 ± 286 mm^3^, while administration of finotonlimab at 2 and 8 mg/kg resulted in significant inhibition of tumor growth, reducing tumor volumes to 509 ± 91 and 448 ± 111 mm^3^, respectively. The corresponding tumor growth inhibitions (TGI %) were calculated as 75% and 79.3% for doses of 2 mg/kg and 8 mg/kg, respectively. Notably, the dose of 2 mg/kg nearly achieved optimal anti-tumor efficiency after administration. In subsequent experiments, we assessed the antitumor efficacy of finotonlimab, pembrolizumab, and nivolumab using a PD-1 humanized B16F1 melanoma mouse model (Figure 4C,D). The results demonstrated that finotonlimab administration led to significant inhibition of tumor growth, and the tumor volumes in the groups treated with finotonlimab (15 mg/kg), nivolumab (15 mg/kg), and pembrolizumab (15 mg/kg) were comparable on day 19 (*p* > 0.05). Moreover, the tumor growth inhibition (TGI %) observed for finotonlimab in the 15 mg/kg group (61.0%) was more pronounced than that in the 5 mg/kg group (51.6%) (*p* > 0.05), showing dose-dependent antitumor activity.

**Figure 5:**
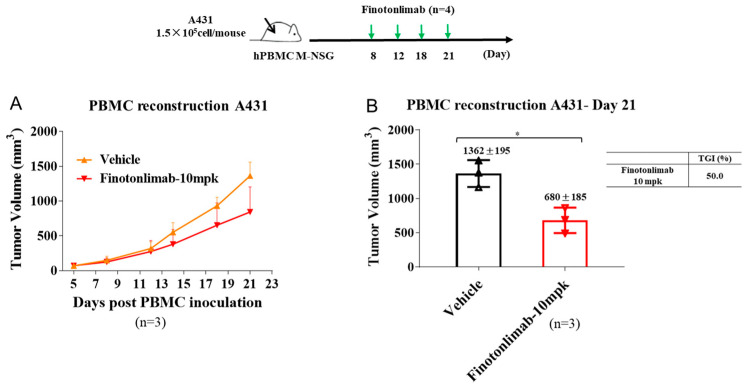
Antitumor activity of finotonlimab monotherapy was evaluated in a PBMC humanized tumor model. The effects of finotonlimab on mouse tumor volume (**A**,**B**) in hPBMC humanized mice (A431 human epidermoid carcinoma). * *p* < 0.05.

To further evaluate the antitumor efficacy of finotonlimab, we utilized an hPBMC-reconstructed mouse model of human A431 cutaneous squamous cell carcinoma, in addition to the hPD-1 humanized model. Mice bearing A431 tumors with consistent initial volumes were administered either vehicle or finotonlimab (10 mg/kg). The finotonlimab-treated group exhibited a decelerated rate of tumor growth than the vehicle-treated group throughout the treatment period. By day 21, the tumor volumes in the finotonlimab and vehicle groups measured 680 ± 185 and 1362 ± 195 mm^3^, respectively, resulting in a TGI % achievement of 50% (Figure 5A,B).

### 2.5. Fc-Mediated Effector Functions of Finotonlimab

**Figure 6:**
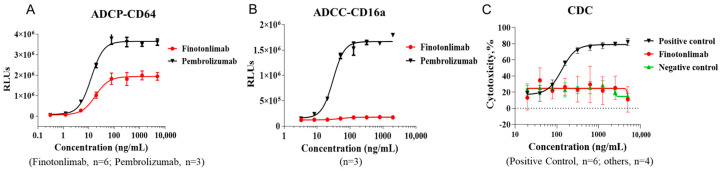
The analog signals of ADCP, ADCC, and CDC for anti-PD-1 antibodies. (**A**) CD64-dependent ADCP mediated by finotonlimab and pembrolizumab (n = 6 and 3, respectively). (**B**) CD16a-dependent ADCC mediated by finotonlimab and pembrolizumab (n = 3/group). (**C**) CDC activity of finotonlimab compared to positive and negative control antibodies (n = 4, 6, and 4, respectively).

Immunoglobulins that bind to cell surface receptors can recruit natural killer (NK) cells, macrophages, and monocytes. Finotonlimab, which contains the human IgG4 subclass, has relatively low binding affinity for human FcγRIII receptors while maintaining binding to human activating FcγRI receptors (CD64s) [19]. The primary role of FcγRI is to activate IgG-bound target cells through antibody-dependent cellular phagocytosis (ADCP). FcγRIIIa (CD16a) serves as the main receptor for NK- and macrophage-mediated antibody-dependent cell-mediated cytotoxicity (ADCC). In this study, we assessed the luciferase signals induced by effector cell-target cell interactions to evaluate the analog functions of Fc receptor-related cytotoxic effects [20,21]. We also further evaluated the binding activity of anti-PD-1 antibodies to CD64- and CD16a-expressing cells using flow cytometry (FACS), as well as C1q protein using ELISA. Although finotonlimab exhibited activating effects on the effector cells (Jurkat-NFAT-Luc2p-CD64) and demonstrated binding activity to FcγRI reconstructed cells, these effects were lower compared to pembrolizumab (Figure 6A, Supplementary Figure S2A). Conversely, no luciferase signals were observed in effector cells (Jurkat-NFAT-Luc2p-CD16a) when exposed to finotonlimab (Figure 6B), which is significantly lower than that observed with pembrolizumab. Furthermore, finotonlimab demonstrated lower binding activity with Jurkat-CD16A (F158) compared to pembrolizumab (Supplementary Figure S2B). Moreover, both the complement-dependent cytotoxic (CDC) assay and the binding assay with normal human serum complement negative reaction results (Figure 6C, Supplementary Figure S2C), indicating that finotonlimab did not induce CDC activity. These findings suggest that finotonlimab exhibits the differentiated Fc characteristics of the IgG4 isotype, minimizing the killing or phagocytoses of activated T cells, possibly via NK or macrophages in vivo.

### 2.6. Comparative Pharmacokinetics of Finotonlimab and Anti-PD-1 Antibodies in Cynomolgus Monkeys

**Figure 7:**
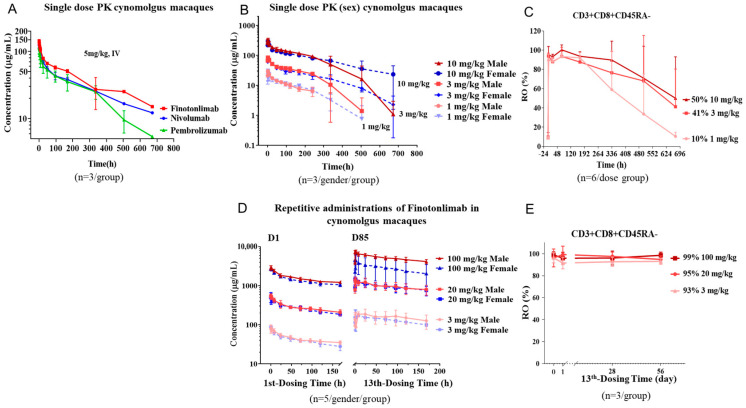
PD-1 receptor occupancy and pharmacokinetic characteristics. (**A**) Serum concentration-time profiles in serum following single intravenous administration of finotonlimab, nivolumab, or pembrolizumab to cynomolgus monkeys. (**B**) Concentration-time profiles in serum after single intravenous administration of different dose of finotonlimab to cynomolgus monkeys. (**C**) PD-1 receptor occupancy profiles on CD3^+^CD8^+^CD45RA^−^ cells after single administration of different doses of finotonlimab. (**D**) Serum concentration-time profiles following repeated administrations of finotonlimab in cynomolgus monkeys (once weekly for 13 doses). (**E**) PD-1 receptor occupancy following the final administration (13th) of different doses of finotonlimab.

The pharmacokinetic profile of pembrolizumab and nivolumab has been proposed to correlate with their pharmacology and efficacy [22,23]. In this study, we investigated the pharmacokinetics of these anti-PD-1 monoclonal antibodies (mAbs) in cynomolgus monkeys following a single intravenous (IV) administration at a dose of 5 mg/kg. Standard pharmacokinetic (PK) measurements revealed serum concentrations indicating a half-life (T_1/2_) of 206 h for finotonlimab, 151 h for pembrolizumab, and 198 h for nivolumab (Figure 7A). Notably, the C_max_ level of finotonlimab was markedly elevated compared to that of pembrolizumab and nivolumab.

### 2.7. Pharmacokinetic and Pharmacodynamic Profiles of Finotonlimab in Cynomolgus Monkeys Following Single and Repeated Administrations

Following a single intravenous (IV) administration of different doses of finotonlimab (1, 3, and 10 mg/kg) in cynomolgus monkeys, the serum concentration-time curves of all dose treatments were depicted according to gender in Figure 7B. The ratio of systemic exposure of finotonlimab (AUC_inf_ and C_0_) increased more than the corresponding dosage proportions as the dose escalated from 1 to 10 mg/kg (Table 2). Initially, there were no gender differences observed in the concentration-time curves of finotonlimab during the initial 120 h at the same dose. However, such differences emerged over time, which is possibly attributable to the development of anti-drug antibodies (ADAs). At day 29 post intravenous administration (IV), all cynomolgus monkeys in the groups receiving doses of 3 and 10 mg/kg tested positive for ADA (Table S1). Following a single intravenous injection of finotonlimab, the PD-1 receptor occupancy (RO) rates of drug on non-naïve CD8^+^ T cells achieved saturation in all animals across all groups, maintaining stability from 2 to 168 h (Figure 7C, Table 3). Subsequently, a gradual decrease in RO % has been observed with a dose-dependent relationship. At 672 h, the mean RO % values for groups receiving doses of 1, 3, and 10 mg/kg were 10.43% (range from 6.19% to 16.75%, n = 6), 41.25% (range from 8.31% to 92.83%, n = 6), and 50% (range from 8.3% to 95.38%, n = 6), respectively.

In the repeated administration study, the serum drug concentrations of finotonlimab were evaluated across three doses (3, 20, and 100 mg/kg) based on gender differences. The peak serum concentrations of finotonlimab exhibited dose-dependent relationships (Table S2, Figure 7D). Notably, significant accumulation of finotonlimab was observed across all groups. The AUC after final administration was higher than that after initial administration. The accumulation indices (AIs) (AUC_last_ (0–168 h) on day 85 to AUC_last_ (0–168 h) on day 1) were 1.45, 3.51 and 3.33 in males and 2.63, 3.63, and 2.03 in females, respectively (Table S2). Furthermore, C_max_, AUC_last_, and AUC_inf_ were higher in males compared to females. These differences were consistent with the pharmacokinetics data obtained from cynomolgus monkeys (Figure 7B). Following the thirteenth administration in cynomolgus monkeys across all dosage groups, the finotonlimab group reached saturation for RO performance, as shown by consistent PD-1 receptor occupancy exceeding 93% for up to eight weeks (Figure 7E). The consistent maintenance of high receptor occupancy throughout repeated administrations suggests that it is possible to achieve saturated receptor occupancy even at a low dose of finotonlimab treatment.

### 2.8. Toxicology Evaluation Following Repeated Administrations of Finotonlimab in Cynomolgus Monkeys

The toxicology profile of finotonlimab was further assessed in cynomolgus monkeys. Following 13 weeks of weekly intravenous dosing, finotonlimab demonstrated excellent tolerability, with no mortality or morbidity observed across all dose groups (3, 20, or 100 mg/kg). Moreover, no apparent treatment-related abnormalities were detected across various parameters, including clinical observations, body weight, body temperature, ECG (electrocardiograph), blood pressure, ophthalmoscopic examinations, coagulation function, blood biochemistry, urinalysis, and organ weights in all groups. The inflammatory cell infiltration detected in multiple organs was attributed to the pharmacological action of finotonlimab and reversed during an 8-week recovery period. Consequently, the NOAEL (no observed adverse effect level) for finotonlimab is considered to be at least 100 mg/kg or higher.

## 3. Discussion

This study provides a comprehensive functional characterization of finotonlimab, a humanized anti-PD-1 monoclonal drug of the IgG4 subtype, including in vitro T cell activation effects, in vivo anti-tumor activity in mouse models, Fc-mediated effector functions, and PK, RO, and safety properties of finotonlimab after single or successive doses in cynomolgus monkeys. These findings indicate that finotonlimab demonstrates a potent and well-tolerated anti-PD-1 profile, and the treatment of finotonlimab could be pursued in clinical trials with relatively low doses and longer administration intervals. Now, the favorable anti-tumor efficacy of finotonlimab has been demonstrated in Phase III trials in several solid tumor types.

Finotonlimab specifically binds to human PD-1 receptors with high affinity (K_D_ = 64.8 pM) and effectively blocks the interaction between PD-1 and its ligands PD-L1 and PD-L2, which resulted in potent T cell activation, characterized by substantial increases in IL-2 and IFN-γ production with a stimulatory effect on CD4^+^ T cells in MLR assays. It is worth mentioning that the IL-2 levels stimulated by finotonlimab were significantly greater compared to nivolumab at the high doses, while the IFN-γ levels stimulated by finotonlimab were significantly higher than those of nivolumab at the lower doses (Figure 3B,C). This differentiation may support a potentially better effect of finotonlimab on T cell activation. Finotonlimab demonstrated robust anti-tumor activity in hPBMC-reconstructed mice bearing A431 xenografts, a model that most closely mimics the distinctive characteristics of the human immune system and tumor microenvironment. In another immune-competent mouse model of B16F1 melanoma, treatment with finotonlimab at 15 mg/kg resulted in a pronounced anti-tumor effect against B16F1 tumors, similar to that observed with nivolumab and pembrolizumab. These studies highlight the favorable biofunctional attributes of finotonlimab as a checkpoint inhibitor and support the potential for robust anti-tumor efficacy of finotonlimab in ongoing clinical trials [24].

Another critical factor in evaluating the effector T cell functions and safety profile of a new anti-PD-1 therapy is cytotoxicity mediated by the binding to complement Fcγ receptors [25,26]. Evidence has shown that anti-PD-1 antibodies must minimize Fc-mediated effector functions (ADCC, ADCP, and CDC) to prevent the unintended killing of PD-1^+^ T cells by FcγR^+^ effector cells [19,27]. In this study, the Fc-mediated effector activity was measured using the robust and reliable recombinant ADCC/P reporter cells, for which sensitivity was higher than for classical NK killing and macrophage cytotoxicity. In accordance with its IgG4 framework, finotonlimab exhibited weak CD16 activation signals and CDC cytotoxicity [19,25,28]. Nevertheless, comparative analyses revealed that finotonlimab induced lower activations by FcγRI (CD64) and FcγRIII (CD16) compared to pembrolizumab, which may be caused by the antibody orientations between PD-1 and Fc receptor-expressing cells. Therefore, finotonlimab is not expected to induce significant depletion of anti-tumor effector T cells [29,30,31].

As earlier discussed, PD-1 monoclonal antibodies block immune-suppressing ligands from interacting with PD-1, thereby enhancing T cell-mediated immune responses [32,33,34]. Consequently, high and sustained PD-1 receptor occupancy is likely to drive more pronounced anti-tumor effects and robust immune responses [33]. Following single intravenous administration of finotonlimab in cynomolgus monkeys, the drug achieved saturation of hPD-1 occupancy on T cells and maintained a slow dephasing rate over an extended period, despite declining serum drug levels. This profile suggests a prolonged duration of therapeutic effect. High levels of PD-1 occupancy on circulating T cells following successive administrations of finotonlimab at different doses were consistently observed for up to 8 weeks following the final administration in each group. It is noteworthy that even 1 mg/kg of finotonlimab in cynomolgus monkeys achieved receptor occupancy exceeding 93%.

Moreover, the possible PK advantage of anti-PD-1 antibodies may correlate with enhanced pharmacodynamics and therapeutic efficacy [23,33,35]. The single-dose PK study demonstrated that finotonlimab exhibited superior pharmacokinetic characteristics than both pembrolizumab and nivolumab at the same dose in cynomolgus monkeys. Following repeated dosing of finotonlimab in cynomolgus monkeys, the increasing ratio of drug exposure (AUC_0–last_ and C_0_) in all groups was higher than that of dose, as shown in Table S2. The same trend was observed in a single-dose PK study of finotonlimab in Table 2. AUC_(0–168h)_ of last administration was higher than that of the first administration with obvious drug accumulation (Table S2). The sustained high PK–RO profile of finotonlimab in animals suggests the potential for durable pharmacodynamics and enhanced immune responses in humans.

Immunogenicity assessment is a crucial metric in the clinical development of antibody-based drugs. In the single-dose PK study, the clearances of 3 and 10 mg/kg groups in the later releasing period were faster than that of the 1 mg/kg group, indicating a non-linear pharmacokinetic profile (Figure 7B). To assess whether decreased PK was due to ADA effects in middle and high dose groups, ADA was detected, with some animals testing positive across all groups (Tables S1 and S3). However, further analysis found that the proportions of ADA at different doses were similar and the titers were all low, so it was speculated that ADA did not affect PK (Table S1). The occurrence of ADA against PD-1 antibodies has been frequently documented in several Food and Drug Administration-approved products, and finotonlimab showed similar ADA characteristics to other anti-PD-1 drugs [36,37]. We will further confirm in clinical trials whether ADA is produced and, if so, whether it affects PK, anti-tumor efficacy and safety, etc.

In summary, finotonlimab, a novel, humanized anti-PD-1 monoclonal antibody, demonstrates high affinity for PD-1 with lower Fc-mediated function and exhibits a durable PK/PD profile and significant in vivo anti-tumor activity. The preclinical findings are encouraging and provide a foundation for evaluating its efficacy and safety in clinical trials.

## 4. Materials and Methods

### 4.1. Reagents

Finotonlimab was produced by SinoCellTech (Beijing, China). Nivolumab was obtained from Bristol-Myers Squibb (New York, NY, USA). Keytruda-biosimilar (anti-PD1 (MK)-IgG4) was sourced from Sino Biological (Beijing, China). Recombinant proteins B7-H1-Fc (PD-L1), PD-1-his, CD28-Fc, CTLA4-His, BTLA-Fc, PIGF-Fc, Kappa-R002/HRP, PD-1 wt/mutant proteins, rhIL-4 (GMP-11846-HNAE), B7-H1-His-biotin, PD-L2-His-biotin, PD-1-Fc, the secondary antibody of goat anti-Human IgG Fc/HRP, the negative control (H7N9-R1-IgG4), and the positive control (PD1-H944-1-IgG1(o), 20171009) were also purchased from Sino Biological (Beijing, China). Goat anti-Human IgG F(ab)2 /HRP was purchased from JACKSON (West Grove, PA, USA). Streptavidin/HRP was obtained from ZSGB-BIO (Beijing, China), while Streptavidin Alexa Fluor^®^ 488 Conjugate was purchased from Life Technologies (Carlsbad, CA, USA). The secondary antibody of PE Mouse Anti-Human CD279 and FITC Mouse Anti-Human CD4 (555346) were purchased from BD Biosciences (Franklin Lakes, NJ, USA). rhGM-CSF (215-GM-010) was obtained from R&D Systems (Minneapolis, MN, USA). Human IFN-γ ELISA Set (555142) and Human IL-2 ELISA Set (555190) were purchased from BD OptEIA^TM^ (Franklin Lakes, NJ, USA). CD4 microbeads (130-045-101), anti-FITC microbeads (130-148-701), and LS Columns (130-042-401) were purchased from Miltenyi Biotec (Bergisch Gladbach, Germany). Ficoll-PaqueTM PLUS (17144002) was sourced from GE Health (Chicago, IL, USA). Luciferase Assay System (E1501) and Passive Lysis 5 × Buffer (E1941) were purchased from Promega (Madison, WI, USA). C1q recombinant protein (A400) was purchased from QuidelOrtho Corporation (San Diego, CA, USA) and its secondary anti C1q/HRP (ab46191) from Abcam (Cambridge, UK).

### 4.2. Animals

Human PD-1 knock-in C57BL/6 mice (B-hPD-1/C57BL/6) were obtained from Biocytogen Pharmaceuticals Co., Ltd. (Beijing, China). Animal procedures adhered to the regulatory standards for laboratory animal care and use at Biocytogen Pharmaceuticals and were approved by the Institutional Animal Care and Use Committee. The mice were maintained under specific pathogen-free (SPF) conditions.

### 4.3. Affinity Measurements by Octet

Biotinylated recombinant human PD-1 protein was loaded using SA sensor (Pall Corporation, New York, NY, USA). Six different concentrations of finotonlimab and nivolumab were tested for real-time association and dissociation analysis using the Octet system. Data were processed and analyzed using Data Analysis Octet software (Data Analysis 6.4).

### 4.4. Epitope Mapping of PD-L1/PD-L2 and Finotonlimab Binding

96-well plates were coated (100 μL/well) with PD-1 wt/mutant proteins diluted to 1 μg/mL at 4 °C overnight. After washing, the plates were blocked for over 1 h at room temperature. Then PD-L1-Fc/PD-L2-Fc or finotonlimab/negative antibodies were diluted to 10 μg/mL and 100 μL/well was added for 2 h. After washing out the free antibodies, 250 ng/mL of IgG Fc/HRP was added at 70 μL/well and incubated for 1 h at room temperature, before the wells were washed. Finally, a chromogenic reaction assay was conducted followed by absorbance measurements (450 nm) in an ELISA reader.

### 4.5. Structural Modeling of Finotonlimab/PD-1 Complex and Epitope Analysis

The structure of finotonlimab was homology modeled by Discovery Studio 4.0 (Accelrys Software), while the structure of PD-1 was derived from the crystal structure (PDB ID 5GGS). The PD-1/finotonlimab complex structure was constructed by docking these structures in Discovery Studio 4.0, guided by the mutation experiment data. The docked complex structures were optimized by energy minimization and the structure with the lowest free energy was selected. For the binding interface analysis, residues of PD-1 within 5.0 Å of finotonlimab (modeled complex structure), PD-L1 (PDB ID 4ZQK), or PD-L2 (PDB ID 6UMT) were recognized as the binding area and visualized using the PyMOL Molecular Graphics System (Version 2.4, Schrödinger, LLC, New York, NY, USA).

### 4.6. Binding Affinity of Finotonlimab by ELISA

96-well plates were pre-coated (100 μL/well) with different concentrations of human PD-1 protein (0.16, 0.49, 1.48, 4.44, 13.33, 40, 120, 360, 1080, 3240, and 9720 ng/mL), human PD-1-His/CD28-Fc/CTLA4-His/BTLA-Fc/PIGF-Fc (1.25, 5, 20, 80, 320, 2560, and 10,240 ng/mL), or anti-PD-1 antibodies at 4 °C overnight. After washing, the plates were blocked for over 1 h at room temperature, then finotonlimab, nivolumab, and H7N9-R1-IgG4 negative control or C1q recombinant protein were added (2 μg/mL or 5 μg/mL, 100 μL/well). Detection used HRP-labeled goat anti-human IgG antibody or secondary anti-C1q/HRP antibody and TMB substrate and the absorbance was read at 450 nm. Origin 7 software was used to calculate EC_50_ and draw profiles.

### 4.7. Blocking Capability of Finotonlimab by ELISA

ELISA was used to evaluate the competitive blocking capability of the testing antibody to PD-L1/PD-L2 against PD-1. Briefly, 96-well plates pre-coated with human PD-1-mFc were incubated with 100 μL 1 μg/mL B7-H1-His or PD-L2-His-biotin and different concentrations (0.003, 0.008, 0.025, 0.074, 0.222, 0.667, 2, 6, 18 μg/mL) of finotonlimab or nivolumab or H7N9-R1-IgG4 negative control. Inhibition was assessed using streptavidin/HRP and TMB substrate. The absorbance was read at 450 nm. The inhibition rate was analyzed as PI% = (OD_blank_ − OD_sample_)/OD_blank_ × 100%. OD_blank_ refers to the negative control group, OD_sample_ refers to the antibody group.

### 4.8. Binding of the Finotonlimab to PD-1 Cell Surfaces by FACS

Jurkat/PD-1 was incubated with anti-PD-1 antibodies or the negative control (2-fold serially diluted from 200 μg/mL to 0.195 μg/mL) at 4 °C for 20 min. After rinsing with PBS and centrifuged, a constant concentration of FITC-labeled Goat anti-human IgG Fc was added and incubated at 4 °C for 20 min. Cells were resuspended in 200 μL of PBS, filtered, and analyzed by flow cytometry to measure mean MFI. The methods used for the finotonlimab cell binding assay to the surface of CD16a (Jurkat-NFAT-Luc2p-CD16A) and CD64 (Jurkat-NFAT-Luc2p-CD64) reconstructed cells were the same as above.

### 4.9. Evaluating Human PD-1/PD-L1 Blocking Capability of Finotonlimab by FACS

The CHO cells expressing human PD-1 were incubated with PD-1 antibodies or the negative control (2-fold serially diluted from 10 μg/mL to 0.26 μg/mL) at 4 °C for 20 min. B7H1-Fc-biotin was added and followed by streptavidin-Alexa Fluor^®^ 488. After rinsing, the steps were the same as above. Inhibition rate was calculated as PI% = (MFI_blank_ − MFI_Sample_)/MFI_blank_ × 100%. OD_blank_ refers to the negative control group, OD_sample_ refers to the antibody group.

### 4.10. T Cell Activation Assay-Associated Luciferase Reporter System

The activation of Jurkat T cells by anti-PD-1 treatment was assessed using a Jurkat PD-1/PD-L1 reporter system, as previously described [38]. Target cells (CHO-K1-PD-L1-CD3E) were seeded at 2 × 10^4^ cells/well in 10% FBS DMEM and cultured overnight. After removing the supernatant, various concentrations of PD-1 antibodies were added. Effector cells (Jurkat-NFAT-Luc2p-PD-1) were dispensed into CHO-K1 cells at 7.5 × 10^4^ cells/well, and the plates were incubated at 37 °C with 5% CO_2_ for 6 h. After incubation, Passive Lysis 5× Buffer (20 μL/well) was added, mixed thoroughly, and 20 μL of supernatant was transferred to a clean 96-well plate. Luminescence was measured using a microplate spectrophotometer within 15 min.

### 4.11. In Vitro Mixed Lymphocyte Reaction

CD4^+^ T cells were isolated from the PBMCs of a healthy donor and dendritic cells were derived from monocytes of a separate donor. Monocytes were cultured for 3 days in RPMI 1640 medium containing 10% FBS, rhGM-CSF (20 ng/mL) and rhIL-4 (160 ng/mL), with half of the culture medium replaced every time. After 6 days, the dendritic cell suspensions were collected. Dendritic cells (1 × 10^4^ cells/well) were co-cultured with allogeneic CD4^+^ T cells (1 × 10^5^ cells/well) in the presence of various concentrations (0.01, 0.1, and 1 μg/mL) of anti-PD-1 antibodies or isotype control antibodies for 5 days. T cell activation was assessed by measuring the level of IL-2 and IFN-γ in supernatants using ELISA.

### 4.12. ADCC/ADCP-Activation Assays

As previous described, FcγR functions were confirmed using a reporter system. For the ADCC (CD16a) or ADCP (CD64) assays, the target cells (CHO-PD-1) were seeded in 96-well plates and cultured overnight in DMEM medium with 10% FBS. Cells were washed with 0.5 g/L PF68-contained RPMI 1640 (Phenol Red-free), then incubated with effector cells (Jurkat-NFAT-Luc2p-CD16a or Jurkat-NFAT-Luc2p-CD64) and different concentrations of antibodies (40 μL/well) for 4 h. After incubating, passive lysis 5 × Buffer with 20 μL/well was added and mixed well. Then the absorbance was measured using a microplate spectrophotometer.

### 4.13. CDC Cytotoxicity Assay

96-well plates were seeded with CHO-PD-1 (5 × 10^4^ cells/well) in 0.1% BSA-contained 1640 culture medium (Phenol Red-free). Then different concentrations of antibodies (50 μL/well) were mixed with the complement (50 μL/well, 1:4 dilution) and incubated at 37 °C for 3 h. WST-8 substrate (15 μL) was added and the absorbance was read using a microplate spectrophotometer.

### 4.14. MC38 Tumor Mouse Models

The mouse colon carcinoma MC38 cells [39] were subcutaneously inoculated to the right flank of the hPD-1 knock-in mice (5 × 10^5^ cells/mouse). When tumors reached about 150 mm^3^, the mice were randomly divided into three groups: vehicle, 2 mg/kg of finotonlimab, and 8 mg/kg of finotonlimab (n = 8/group, i.p.). Mice were administered once every 3 days for five doses. Tumor volume, tumor growth inhibitory rate (TGI %), and body weights were measured twice a week until day 19. Mice were euthanized 7 days after the final dose, and tumor tissues were weighed and frozen at −80 °C for further analysis.

### 4.15. B16F1 Tumor Mouse Models

The mouse melanoma B16F1 cells [40] were subcutaneously inoculated to the right side of the backs of the hPD-1 knock-in mice (1 × 10^6^ cells/mouse). When tumors reached about 100~150 mm^3^ (3 days after cell seeding), mice were randomly divided into five groups: vehicle group, 5 mg/kg of finotonlimab group, 15 mg/kg of finotonlimab group, 15 mg/kg of pembrolizumab group, and 15 mg/kg of nivolumab group (n = 10/group, i.p.). Mice were treated twice weekly for 2 weeks. Tumor volumes and TGI % were measured twice a week. Mice were euthanized 5 days after the last administration, and tumor tissues were weighed and frozen at −80 °C for further analysis.

### 4.16. hPBMC-Reconstituted A431 Mouse Models

A431 cells (1.5 × 10^5^ cell/mouse) were subcutaneously injected into the right side of the backs of M-NSG mice. The next day, hPBMC cells (100 μL, 5 × 10^6^ cells) were injected intravenously via tail vein [41]. When tumors reached about 100~150 mm^3^, mice were randomly divided into vehicle group and 10 mg/kg of finotonlimab group (n = 3/group, i.p.). The mice were treated twice weekly for 2 weeks. Tumor volume and TGI % were measured post-treatment. Tumor tissues were weighed and frozen at −80 °C for further analysis.

### 4.17. Pharmacokinetic and Toxicity Assessments of Finotonlimab in Cynomolgus Monkeys

In a single-dose pharmacokinetic study, cynomolgus monkeys received finotonlimab at 1, 3, and 10 mg/kg (2/gender/group) by single intravenous administrations. Cynomolgus monkeys (nine males and nine females) were randomly assigned to three dose groups. Blood samples for PK assessments were collected before (0) dosing and at 0.042, 1, 2, 8, 24, 48, 72, 96, 120, 168, 240, 336, 504, and 672 h post-dosing. Hematological and biochemical tests were conducted on the 2nd, 8th, and 15th days after administration. Animals were euthanized after a two-week observation period and tissues were examined histopathologically.

In a 13-week toxicology study, cynomolgus monkeys (5/gender/group) received 13 successive weekly IV doses of finotonlimab at 3, 20, and 100 mg/kg, administered at a constant volume of 0.4 mL/kg followed by an 8-week recovery period. Safety assessments included general clinical observations, body weight, food consumption, body temperature, electrocardiogram, blood pressure, blood oxygen saturation, ophthalmoscopic examinations, hematology, coagulation, clinical chemistry, urinalysis, lymphocyte subsets, C-reactive protein, serum complement, the specific IgG antibodies and nAb, organ weights, and macroscopic and microscopic examinations. Three monkeys/gender of each group were euthanized at 1 week (D92) after the last administration and the remaining animals were euthanized at the end of the recovery period (D141).

The sandwich ELISA method was used to establish a drug concentration detection and analysis method for SCT-I10A in serum for cynomolgus monkeys and systematic methodological validation was completed, including selectivity, specificity, lower limit of quantitation (LLOQ), upper limit of quantitation (ULOQ), standard curve, precision, accuracy, stability, dilution linearity, hook effect and parallelism. The quantification was ranged from 0.9–30 μg/mL.

### 4.18. Anesthesia and Euthanasia

Anesthesia procedures were carried out in accordance with AVMA Guidelines for the Euthanasia of Animals (the American Veterinary Medical Association, 2013). All animals were sedated with intramuscular injections of ketamine (10 mg/kg and 50 mg/mL) at the endpoint, followed by intravenous injections of pentobarbital sodium (20 mg/kg and 20 mg/mL) for anesthesia, followed by femoral artery bleeding for euthanasia. In the single-dose pharmacokinetic study, all animals were euthanized at the end of the two-week observation period. In the toxicology studies, three monkeys/gender of each group were euthanized at 1 week (D92) after the last administration and the remaining animals were euthanized at the end of the 8-week recovery period (D141). In the anti-tumoral mice model, all mice were euthanized at the endpoints of the experiments. During the experiments, when the tumor volumes reached above 2000 mm^3^, the mice were euthanized to minimize suffering and distress.

### 4.19. Receptor Occupancy Assay

The whole blood sample was collected at 2, 24, 72, 168, 336, 504, and 672 h after the first administration for PK assessment. The receptor occupancy assay was measured by FACS.

Erythrocytes were lysed by incubating 700 μL of whole blood with 7 mL of lysing buffer for 15 min at room temperature. Then erythrocytes were washed with PBS, centrifuged at 300× *g* for 5 min at 25 °C, and resuspended in 300 μL of PBS. The cell suspension was then divided into four tubes (A, B, C, and D).

For staining and incubation, anti-PD-1 antibodies or H7N9 antibodies were added into the tubes of C and D and the mixture was vortexed and incubated at 4 °C for 20 min. Next, 3 mL of PBS was added to each of tubes A, B, C, and D. The cell suspension was centrifuged at 1400× *g* rpm for 5 min and the supernatant was removed. The cells were washed by adding 3 mL of PBS buffer, followed by centrifugation at 1400× *g* rpm for 5 min with the supernatant removed. Human IgG antibodies were added into the tubes of B, C, and D. The tubes were vortexed and incubated at 4 °C for 15 min. Each tube was then washed twice with 3 mL of washing buffer and centrifuged at 1400× *g* rpm for 5 min with the supernatant removed. The antibodies of CD3, CD4, CD8, and CD45RA were then added, vortexed and incubated at 4 °C for 20 min. The cells were washed twice again with 3 mL of washing buffer and centrifuged at 1400× *g* rpm for 5 min. After filtering the cell suspension through a cell strainer, receptor occupancy was measured by FACS assay.

### 4.20. Statistical Analysis

The results were analyzed using means and standard errors (Mean ± SEM), plotted using GraphPad Prism 5.01, and subjected to statistical *t*-tests using Excel. *p* < 0.05 indicates a significant difference.

## 5. Conclusions

This study has revealed a humanized anti-PD-1 antibody tested in Phase III trials for several solid tumor types, finotonlimab, effectively enhances human T cell function in vitro and exhibits significant antitumor efficacy in vivo using both PD-1 humanized and PBMC-reconstructed mouse models. Furthermore, finotonlimab exhibited limited impact on Fc receptor-dependent effector cell activation and exhibited a nonlinear pharmacokinetic (PK) profile in cynomolgus monkeys, which was better than that of marketed antibodies (nivolumab or pembrolizumab). All these preclinical findings are promising and lay the groundwork for evaluating the therapeutic potential and pharmacodynamic properties of finotonlimab in clinical trials.

## Figures and Tables

**Table 1:** Comparative binding affinity of finotonlimab and nivolumab.

	K_D_ (M)	K_on_ (M^−1^s^−1^)	K_dis_ (s^−1^)
	7.13 × 10^−11^	2.95 × 10^5^	2.10 × 10^−5^
Finotonlimab	5.46 × 10^−11^	3.06 × 10^5^	1.67 × 10^−5^
	6.84 × 10^−11^	3.03 × 10^5^	2.07 × 10^−5^
Mean	6.48 × 10^−11^	3.01 × 10^5^	1.95 × 10^−5^
	6.80 × 10^−11^	6.89 × 10^5^	4.68 × 10^−5^
Nivolumab	7.92 × 10^−11^	7.02 × 10^5^	5.56 × 10^−5^
	7.50 × 10^−11^	6.84 × 10^5^	5.13 × 10^−5^
Mean	7.41 × 10^−11^	6.92 × 10^5^	5.12 × 10^−5^

**Table 2:** PK characteristics of finotonlimab following single intravenous administrations of 1, 3, and 10 mg/kg to cynomolgus monkeys (n = 3/gender/group).

Dose (mg/kg)	Gender	/	T_1/2_ h	C_max_ μg/mL	AUC_last_ h·mg/mL	AUC_inf_ h·mg/mL	MRT h	C_max_ Ratio	AUC Ratio
1	M	Mean	178.34	30.13	2.71	4.46	92.09	1.00	1.00
		SD	45.79	2.59	0.37	1.25	6.06		
	F	Mean	128.50	25.02	3.44	3.87	136.68	1.00	1.00
		SD	25.43	9.95	0.85	0.91	25.12		
3	M	Mean	169.15	84.58	10.89	14.40	132.52	2.81	4.02
		SD	26.36	7.20	2.30	1.58	34.03		
	F	Mean	202.20	84.28	12.53	14.23	194.32	3.37	3.64
		SD	41.68	5.71	1.23	2.47	6.66		
10	M	Mean	191.71	339.39	44.22	61.49	151.24	11.26	16.33
		SD	44.67	33.15	10.76	5.38	62.38		
	F	Mean	215.62	299.93	49.72	60.24	217.85	11.99	14.45
		SD	154.11	27.57	10.02	20.85	48.02		

**Table 3:** PD-1 receptor occupancy of finotonlimab following a single intravenous injection of 1, 3, and 10 mg/kg to cynomolgus monkeys (n = 6/group).

Receptor Occupation	1 mg/kg		3 mg/kg		10 mg/kg	
Time for Blood Collection	Mean (%)	SD (%)	Mean (%)	SD (%)	Mean (%)	SD (%)
Before dose	8.65	2.03	8.94	2.46	9.67	4.76
2 h	89.52	6.60	93.90	3.11	95.35	8.51
24 h	88.47	4.38	87.98	3.87	93.17	2.61
72 h	93.33	2.79	93.52	9.54	100.36	5.07
168 h	92.09	5.44	87.68	4.65	93.69	4.63
336 h	59.00	40.16	76.51	22.26	89.80	19.64
504 h	33.68	41.48	68.09	47.21	70.91	33.26
672 h	10.43	4.32	41.26	39.19	50.00	43.11

## Data Availability

The data generated in this study have not been deposited into any publicly available repository. All the data are included in the article and Supporting Material.

## Supplementary Materials

The following supporting information can be downloaded at: https://www.mdpi.com/article/10.3390/ph18030395/s1.

## References

[1] Lee J.J., Chu E. (2018). Recent Advances in the Clinical Development of Immune Checkpoint Blockade Therapy for Mismatch Repair Proficient (pMMR)/non-MSI-H Metastatic Colorectal Cancer. Clin. Color. Cancer.

[2] Centanni M., Moes D., Trocóniz I., Ciccolini J., Van Hasselt J.G.C. (2019). Clinical Pharmacokinetics and Pharmacodynamics of Immune Checkpoint Inhibitors. Clin. Pharmacokinet..

[3] Parvez A., Choudhary F., Mudgal P., Khan R., Qureshi K.A., Farooqi H., Aspatwar A. (2023). PD-1 and PD-L1: Architects of immune symphony and immunotherapy breakthroughs in cancer treatment. Front. Immunol..

[4] Ishida Y., Agata Y., Shibahara K., Honjo T. (1992). Induced expression of PD-1, a novel member of the immunoglobulin gene superfamily, upon programmed cell death. EMBO J..

[5] Yao S., Chen L. (2014). PD-1 as an immune modulatory receptor. Cancer J..

[6] Munari E., Mariotti F.R., Quatrini L., Bertoglio P., Tumino N., Vacca P., Eccher A., Ciompi F., Brunelli M., Martignoni G. (2021). PD-1/PD-L1 in Cancer: Pathophysiological, Diagnostic and Therapeutic Aspects. Int. J. Mol. Sci..

[7] Yasutoshi A., Akemi K., Hiroyuki N., Yasumasa I., Takeshi T., Hideo Y., Honjo T. (1996). Expression of the PD-1 antigen on the surface of stimulated mouse T and B lymphocytes. Int. Immunol..

[8] Yao S., Wang S., Zhu Y., Luo L., Chen L. (2009). PD-1 on dendritic cells impedes innate immunity against bacterial infection. Blood.

[9] Said E.A., Dupuy F.P., Trautmann L., Zhang Y., Sekaly R.P. (2010). Programmed death-1-induced interleukin-10 production by monocytes impairs CD4^+^ T cell activation during HIV infection. Nat. Med..

[10] Dong H., Strome S.E., Salomao D.R., Tamura H., Hirano F., Flies D.B., Roche P.C., Lu J., Zhu G., Tamada K. (2002). Tumor-associated B7-H1 promotes T-cell apoptosis: A potential mechanism of immune evasion. Nat. Med..

[11] Ahmadzadeh M., Johnson L.A., Heemskerk B., Wunderlich J.R., Dudley M.E., White D.E., Rosenberg S.A. (2009). Tumor antigen-specific CD8 T cells infiltrating the tumor express high levels of PD-1 and are functionally impaired. Blood.

[12] Latchman Y., Wood C.R., Chernova T., Chaudhary D., Borde M., Chernova I., Iwai Y., Long A.J., Brown J.A., Nunes R. (2001). PD-L2 is a second ligand for PD-1 and inhibits T cell activation. Nat. Immunol..

[13] Kim J.M., Chen D.S. (2016). Immune escape to PD-L1/PD-1 blockade: Seven steps to success (or failure). Ann. Oncol..

[14] Han Y., Liu D., Li L. (2020). PD-1/PD-L1 pathway: Current researches in cancer. Am. J. Cancer Res..

[15] Almagro J.C., Daniels-Wells T.R., Perez-Tapia S.M., Penichet M.L. (2017). Progress and Challenges in the Design and Clinical Development of Antibodies for Cancer Therapy. Front. Immunol..

[16] Chames P., Regenmortel M.V., Weiss E., Baty D. (2009). Therapeutic antibodies: Successes, limitations and hopes for the future. Br. J. Pharmacol..

[17] Kim J., Lee J.Y., Kim H.G., Kwak M.W., Kang T.H. (2021). Fc Receptor Variants and Disease: A Crucial Factor to Consider in the Antibody Therapeutics in Clinic. Int. J. Mol. Sci..

[18] Shi Y., Guo W., Wang W., Wu Y., Fang M., Huang X., Han P., Zhang Q., Dong P., Zhou X. (2024). Finotonlimab with chemotherapy in recurrent or metastatic head and neck cancer: A randomized phase 3 trial. Nat. Med..

[19] Chen X., Song X., Li K., Zhang T. (2019). FcγR-Binding Is an Important Functional Attribute for Immune Checkpoint Antibodies in Cancer Immunotherapy. Front. Immunol..

[20] Cheng Z.J., Garvin D., Paguio A., Moravec R., Engel L., Fan F., Surowy T. (2014). Development of a robust reporter-based ADCC assay with frozen, thaw-and-use cells to measure Fc effector function of therapeutic antibodies. J. Immunol. Methods.

[21] Uccellini M.B., Aslam S., Liu S.T.H., Alam F., García-Sastre A. (2021). Development of a Macrophage-Based ADCC Assay. Vaccines.

[22] Deng R., Bumbaca D., Pastuskovas C.V., Boswell C.A., West D., Cowan K.J., Chiu H., McBride J., Johnson C., Xin Y. (2016). Preclinical pharmacokinetics, pharmacodynamics, tissue distribution, and tumor penetration of anti-PD-L1 monoclonal antibody, an immune checkpoint inhibitor. MAbs.

[23] Kurino T., Matsuda R., Terui A., Suzuki H., Kokubo T., Uehara T., Hatakeyama H., Hisaka A., Arano Y. (2020). Poor outcome with anti-programmed death-ligand 1 (PD-L1) antibody due to poor pharmacokinetic properties in PD-1/PD-L1 blockade-sensitive mouse models. J. Immunother. Cancer.

[24] Gridelli C., Ardizzoni A., Barberis M., Cappuzzo F., Casaluce F., Danesi R., Troncone G., De Marinis F. (2017). Predictive biomarkers of immunotherapy for non-small cell lung cancer: Results from an Experts Panel Meeting of the Italian Association of Thoracic Oncology. Transl. Lung Cancer Res..

[25] Bruhns P., Iannascoli B., England P., Mancardi D.A., Fernandez N., Jorieux S., Daëron M. (2009). Specificity and affinity of human Fcgamma receptors and their polymorphic variants for human IgG subclasses. Blood.

[26] Junker F., Gordon J., Qureshi O. (2020). Fc Gamma Receptors and Their Role in Antigen Uptake, Presentation, and T Cell Activation. Front. Immunol..

[27] Zhang T., Song X., Xu L., Ma J., Zhang Y., Gong W., Zhang Y., Zhou X., Wang Z., Wang Y. (2018). The binding of an anti-PD-1 antibody to FcγRΙ has a profound impact on its biological functions. Cancer Immunol. Immunother..

[28] Labrijn A.F., Buijsse A.O., van den Bremer E.T.J., Verwilligen A.Y.W., Bleeker W.K., Thorpe S.J., Killestein J., Polman C.H., Aalberse R.C., Schuurman J. (2009). Therapeutic IgG4 antibodies engage in Fab-arm exchange with endogenous human IgG4 in vivo. Nat. Biotechnol..

[29] Swisher J.F., Feldman G.M. (2015). The many faces of FcRI: Implications for therapeutic antibody function. Immunol. Rev..

[30] Nimmerjahn F., Ravetch J.V. (2008). Fcγ receptors as regulators of immune responses. Nat. Publ. Group.

[31] Delidakis G., Kim J.E., George K., Georgiou G. (2022). Improving Antibody Therapeutics by Manipulating the Fc Domain: Immunological and Structural Considerations. Annu. Rev. Biomed. Eng..

[32] Shchelokov D., Demin O. Abstract 2233: Prediction and Comparison of PD-1 Receptor Occupancy in the Tumor after Treatment with Immune Checkpoint Inhibitors. Proceedings of the AACR Annual Meeting.

[33] Wang J., Fei K., Jing H., Wu Z., Wu W., Zhou S., Ni H., Chen B., Xiong Y., Liu Y. (2019). Durable blockade of PD-1 signaling links preclinical efficacy of sintilimab to its clinical benefit. Mabs.

[34] Fu J., Wang F., Dong L.H., Xing M.J., Song H.F. (2019). Receptor occupancy measurement of anti-PD-1 antibody drugs in support of clinical trials. Bioanalysis.

[35] Brahmer J.R., Drake C.G., Wollner I., Powderly J.D., Picus J., Sharfman W.H., Stankevich E., Pons A., Salay T.M., McMiller T.L. (2010). Phase I study of single-agent anti-programmed death-1 (MDX-1106) in refractory solid tumors: Safety, clinical activity, pharmacodynamics, and immunologic correlates. J. Clin. Oncol..

[36] Lou B., Wei H., Yang F., Wang S., Yang B., Zheng Y., Zhu J., Yan S. (2021). Preclinical Characterization of GLS-010 (Zimberelimab), a Novel Fully Human Anti-PD-1 Therapeutic Monoclonal Antibody for Cancer. Front. Oncol..

[37] Kumar S., Ghosh S., Sharma G., Wang Z., Kehry M., Marino M., Neben T.Y., Lu S., Luo S., Roberts S. (2021). Preclinical characterization of dostarlimab, a therapeutic anti-PD-1 antibody with potent activity to enhance immune function in in vitro cellular assays and in vivo animal models. mAbs.

[38] Sharma S. (2020). Pan-TGFβ inhibition by SAR439459 relieves immunosuppression and improves antitumor efficacy of PD-1 blockade. OncoImmunology.

[39] Greenlee J.D., King M.R. (2022). A syngeneic MC38 orthotopic mouse model of colorectal cancer metastasis. Biol. Methods Protoc..

[40] Yamamoto M., Tanaka Y., Takeda R., Nakamoto A., Nakamoto M., Yagita H., Sakai T. (2024). Soy isoflavone genistein attenuates the efficacy of immune checkpoint therapy in C57BL/6 mice inoculated with B16F1 melanoma and a high PD-L1 expression level reflects tumor resistance. J. Clin. Biochem. Nutr..

[41] Zhou Y., Shen H., Wu M., Wang J., Wu Z., Fu F., Liu Y., Lu J., Yao Y., Luo N. (2023). Pharmacology, pharmacokinetics, and toxicity characterization of a novel anti-CD73 therapeutic antibody IBI325 for cancer immunotherapy. Int. J. Biol. Macromol..

