# Supplementary material for: Preclinical Characterization of Efficacy and Pharmacodynamic Properties of Finotonlimab, a Humanized Anti-PD-1 Monoclonal Antibody
Source: Pharmaceuticals (Basel). 2025 Mar 12;18(3):395. doi: 10.3390/ph18030395 (PMC11946465; doi:10.3390/ph18030395)
Supplement: Supplementary file 1 [file pharmaceuticals-18-00395-s001.zip › pharmaceuticals-3496477-supplementary.pdf]

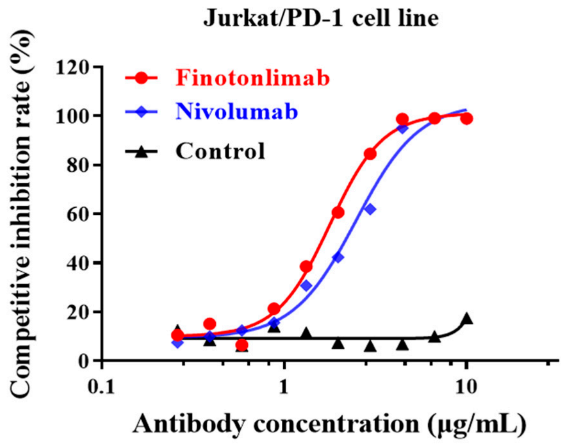

Figure S1. Finotonlimab and Nivolumab competitively block the binding of PD-L1 with PD-1 in Jurkat cells by FACS (n=1).

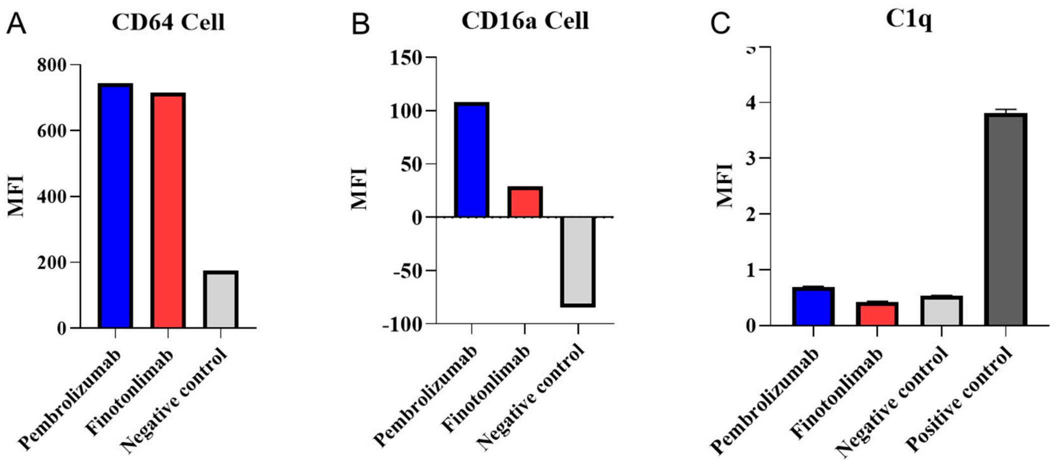

Figure S2. The binding activities of anti-PD-1 antibodies with (A) FcγRI (CD64), (B) FcγRIIIa (CD16a) reconstructed cells and (C) C1q protein.

Table S1. The incidence of ADA and the corresponding titer after single intravenous administration of Finotonlimab at 1, 3, 10 mg/kg in cynomolgus macaques (n=6/group).

Time (day)	Dosage (mg/kg)		
	**1**	**3**	**10**
**D-1**	0/6	0/6	1/6（<2）
**D-15**	5/6（<2~256）	5/6（2~256）	6/6（2~256）
**D-29**	6/6（64~512）	6/6（4~512）	6/6（<1~512）

Table S2. TK parameters after repeat intravenous administrations of Finotonlimab at 3, 20, 100 mg/kg in cynomolgus macaques (n=3/sex).

Dose (mg/kg)	Time	Sex		C_{max} μg/mL	AUC_{last} h*mg/mL	AUC_{inf} h*mg/mL	C_{max} Ratio	AUC_{last} Ratio	AI
3	1st dose (0-168h)	M	Mean	85.05	7.47	30.25	1.00	1.00	
			SD	16.43	0.78	19.42			
		F	Mean	88.13	6.85	16.98	1.00	1.00	
			SD	11.23	0.78	12.57			
	13th dose	M	Mean	96.89	10.82	60.18	1.00	1.00	1.45
			SD	115.87	15.49	22.93			
		F	Mean	165.67	18.04	55.29	1.00	1.00	2.63
			SD	86.84	10.22	9.17			
20	1st dose (0-168h)	M	Mean	556.96	47.06	120.71	6.55	6.30	
			SD	58.30	6.19	29.36			
		F	Mean	528.23	44.42	87.54	5.99	6.48	
			SD	141.09	2.54	7.49			
	13th dose	M	Mean	1437.81	165.14	534.94	14.84	15.26	3.51
			SD	283.03	41.21	255.06			
		F	Mean	1501.59	161.04	472.11	9.06	8.93	3.63
			SD	247.46	30.82	244.05			
100	1st dose (0-168h)	M	Mean	3107.12	263.73	807.69	36.53	35.29	
			SD	299.77	10.27	284.14			
		F	Mean	2806.97	233.82	597.71	31.85	34.12	
			SD	142.08	16.27	179.00			
	13th dose	M	Mean	7729.03	877.23	2565.73	79.77	81.08	3.33
			SD	758.69	95.84	578.56			
		F	Mean	4651.37	474.97	994.90	28.08	26.33	2.03
			SD	1866.54	285.38	721.87			

Table S3. The incidence of ADA and the corresponding titer after repeat doses of Finotonlimab at 3, 20, and 100 mg/kg in cynomolgus macaques (n=10/group).

**Time (day)**	**Dosage (mg/kg)**			
	**Vehicle**	**3**	**20**	**100**
**D-5**	0/10	0/10	2/10（<1~2）	1/10（4）
**D-14**	0/10	6/10（<2~16）	5/10（<1~16）	4/10（<2~8）
**D-28**	0/10	7/10（<1~2048）	7/10（<1~8）	3/10（<1~16）
**D-42**	0/10	7/10（<1~4096）	3/10（2~128）	2/10（<1~256）
**D-56**	1/10（<2）	8/10（<1~4096）	5/10（<1~8）	4/10（<1~8，1024b ）
**D-70**	0/10	8/10（<1~8192）	3/10（<1~64）	3/10（<1~128，2048b ）
**D-84**	0/10	8/10（<1~8192）	3/10（<1~16）	1/10（2048b ）
**D-112**	0/4	2/4（<2~8192）	0/4	1/4（16）
**D-141**	0/4	2/4（2~4096）	1/4（<1）	1/4（8）
